# North Texas Medical Association

**Published:** 1898-06

**Authors:** R. D. Potts, R. F. Miller

**Affiliations:** President, Bonham, Texas; Secretary, Sherman, Texas


### North Texas Medical Association.

BONHAM, TEXAS, May 20, 1898.

DEAR DOCTOR:—The next semi-annual meeting of the North Texas Medical Aseociation will be held in the city of Fort Worth, Texas, beginning Tuesday, June 21st, 1898, and will continue in session for three days. The meeting will be called to order promptly at 10 o'clock a. m.

Your presence on this occasion is earnestly desired. We confidently expect a meeting of unusual pleasure as well as profit. The program contains many papers upon subjects of exceptional interest, and by men who are capable of entertaining us.

The profession of Fort Worth will extend to us their usual hospitality. So you are cordially invited to be present, and enjoy with us what we expect to be one of the best meetings we have ever had.

R. D. POTTS, President, Bonham.

R. F. MILLER, Secretary, Sherman.

